# Multicentre study evaluating the non-inferiority of the new paediatric formulation of artesunate/amodiaquine versus artemether/lumefantrine for the management of uncomplicated *Plasmodium falciparum* malaria in children in Cameroon, Ivory Coast and Senegal

**DOI:** 10.1186/1475-2875-11-433

**Published:** 2012-12-27

**Authors:** Babacar Faye, Thomas Kuété, Christiane P Kiki-Barro, Roger C Tine, Thérèse Nkoa, Jean Louis A Ndiaye, Claude A Kakpo, Khadime Sylla, Hervé EI Menan, Oumar Gaye, Oumar Faye, Albert Same-Ekobo, Koné Moussa

**Affiliations:** 1 Service de Parasitologie-Mycologie, Faculté de Médecine, Université Cheikh Anta Diop, Dakar Fann, Dakar, BP 5005, Sénégal; 2 Faculté de Médecine et Sciences Pharmaceutiques, Université de Douala, BP: 2701, Douala, Cameroun; 3 Laboratoire de Parasitologie-Mycologie, UFR des Sciences Pharmaceutiques et BiologiquesUniversité de Cocody CeDReS, CHU de Treichville, Abidjan, 01 B.P.V 34, Côte d'Ivoire; 4 Faculté de Médecine et Sciences Biomédicales, Université de Yaoundé 1, BP 3266, Yaoundé, Cameroun; 5 Centre de Santé El RAPHA, 13 B.P 3199, Abidjan, Côte d'Ivoire; 6 Centre Hospitalier Universitaire de Yaoundé, BP 3266, Yaoundé, Cameroun

**Keywords:** Malaria, Children, Artesunate plus Amodiaquine, West Africa

## Abstract

**Background:**

This multicentre study was carried out in Cameroon, Ivory Coast and Senegal to evaluate the non-inferiority of the new paediatric formulation of artesunate/amodiaquine (AS+AQ)(Camoquin-Plus Paediatric®) in suspension form versus artemether/lumefantrine (AL)(Coartem®) in the management of African children with uncomplicated falciparum malaria.

**Methods:**

It was an open randomized trial including children aged between 7 months and 7 years. The endpoints were Adequate Clinical and Parasitological Response (ACPR) at day 28, the clinical and biological tolerability. Statistical analyses were done in Intention To Treat (ITT) and in Per protocol (PP).

**Results:**

At the end of the study 481 patients were enrolled in the three countries (249 in the AS+AQ arm and 232 in the AL arm). ACRP in ITT after PCR correction did not show any statistical difference between the two groups with 97.6% for AS+AQ versus 94.8% for AL. In the PP analysis, the corrected ACRP were respectively 98.7% and 96.9% for the two regimens. The clinical tolerance was good without significant difference. Anaemia was significantly higher at D7 in the two groups compared to D0.

**Conclusion:**

This study demonstrates the non-inferiority of AS+AQ versus AL, its efficacy and tolerance in the management of uncomplicated *Plasmodium falciparum* malaria in African children.

## Background

In spite of all the efforts that have been made over decades, malaria remains a major public health problem in sub-Saharan Africa. Children are still the main victims of the disease with a very high mortality rate [1]. Since 2006, the recommendation by WHO is to use artemisinin-based combination therapy (ACT) in response to the rise in resistance to the usual treatment (chloroquine, amodiaquine or sulphadoxine-pyrimethamine) of uncomplicated *Plasmodium falciparum* malaria [2]. Artesunate associated to amodiaquine (AS+AQ) is one of the formulations of ACT recommended and widely used in Africa. A large number of studies showed its efficacy [3]. Unfortunately, most of the formulations of this combination were not suitable for children. In response to this, pharmaceutical firms developed paediatrics formulations more suited to the treatment of children. Thus, Pfizer laboratories developed the combination of artesunate and amodiaquine in suspension form (Camoquin Plus Paediatric®). Few randomized multicentre trials were done with this new formulation. The main focus of this study conducted in 2008 and 2009 in three sites in West Africa (Cameroon, Ivory Coast and Senegal) was to determine the non-inferiority at day 28 of this new AS+AQ paediatric formulation versus artemether/lumefantrine (Coartem®) in the treatment of uncomplicated *P. falciparum* malaria among children aged between six months and seven years.

## Methods

### Study site

This study was conducted in three countries, Cameroon in central Africa, Ivory Coast and Senegal in West Africa. In Cameroon, the study was performed in the urban health centre of Melen in Yaoundé, the capital city. In Ivory Coast, it was done in El Rapha Health Centre of Abidjan, the capital city, and in Senegal in the health district of Kaolack located 200 km from Dakar, the capital city.

### Study population

Children above 6 months and under 7 years of age, who came to the local health centre, with *P. falciparum* parasite density between 1,000 and 100,000/μl in a low transmission area (Senegal) and between 2,000 and 200,000/μl in high transmission areas (Ivory Coast and Cameroon) [4], with the presence of an axillary temperature higher than 37.5°C, ability to swallow drugs per os, were enrolled in the study. Patients presenting severe vomiting, signs of severe malaria [5] or severe malnutrition (children whose weight-for-height was less than 70% of the median NCHS/WHO reference value, with a symmetrical oedema involving the feet), patients with allergy to study drugs or who had used any anti-malarial drug within 28 days prior to enrolment were excluded. Each participant’s guardian gave fully informed written consent prior to enrolment.

### Study procedures

This was a multicentre open, comparative and randomized phase IV trial undertaken in two parallel groups to test non-inferiority between two associations: AS+AQ versus artemether/lumefantrine (AL) in children from six months to seven years. After enrolment, children were weighed and randomized into blocks of 10 to receive one of the two drugs.

The formulations of the two associations were the following: AS+AQ in a box with 1 bottle containing AS in powder dosed a 160mg/80ml to suspend and 1 bottle containing AQ in syrup dosed at 50mg/5ml. Children under two years received 10ml of AQ and 20ml of AS per day and children up to two years received 15ml of AQ plus 25 ml of AS per day, in a single administration.

AL was presented in fixed tablets containing 20mg of artemether and 120 mg of lumefantrine. Tablets were crushed and mixed with water before administration. No food was given prior the AL administration. It was given twice a day according to the patient’s bodyweight and the manufacturer’s instructions. The two drug regimens covered three days (day 0 to day 2). All the daily doses were administered at the health post, under the direct supervision of the investigators. In case of vomiting within the 30 minutes following the administration, study doses were administered again to the patient. Patients who kept vomiting were withdrawn.

Study participants were examined in the study clinic 1, 2, 3, 7, 14, 21, and 28 days after enrolment or at any time if they did not feel well. Quinine treatment was given in case of treatment failure which includes : early treatment failure (development of danger signs or severe malaria on Day 1, Day 2 or Day 3, in the presence of parasitaemia, or parasitaemia on Day 2 higher than Day 0 count irrespective of axillary temperature, or parasitaemia on Day 3 with axillary temperature ≥3 7.5°C, or parasitaemia on Day 3 ≥ 25% of count on Day 0) and late treatment failure (development of danger signs or severe malaria after Day 3 in the presence of parasitaemia, without previously meeting any of the criteria of early treatment failure or presence of parasitaemia and axillary temperature ≥ 37.5°C on any day from Day 4 to Day 28, without previously meeting any of the criteria of early treatment failure) [4].

### Biological follow up

Finger prick to obtain blood for thick and thin smears were done at Day 1, 2, 3, 7, 14, 21 and Day 28 after inclusion or at any time if the patients did not feel well. Blood smears were stained with Giemsa and 200 leucocytes were counted. Assuming a total leukocytes count of 8,000 per litre, parasite density was determined as the number of asexual parasites × 8,000/200 [4]. The slide readers were kept blinded to the treatment allocated.

### Polymerase chain reaction (PCR)

Four drops of blood were collected on filter paper for each patient at day 0 and at day of recurrent parasitaemia to distinguish recrudescence from new infection. For this, parasite genotyping by nested PCR was conducted to compare two polymorphic genetic markers from *Plasmodium falciparum*: Merozoite Surface Protein (*msp*) genes 1 and 2. The primers and schedules described by Faye *et al*[6] were used.

### Tolerability and safety assessment

All adverse events were closely monitored during each scheduled visit by interview with patient if possible or guardian and clinical examination by a physician. All signs noted were reported in the case report form. Adverse events were defined as occurring new events or worsening from baseline after administration of treatment. Haematology and biochemical tests were done at the enrolment and at Day 7 to evaluate haemoglobin, creatinine, aspartate amino transferase (ASAT), alanine amino transferase (ALAT) and bilirubine parameters. Biological abnormalities were noted.

### Data statistical analysis

The number of patients to be included in this study was determined using Epi info software version 6.04d [7], On the basis of previous studies, the cure rate of AL was estimated at 95% [8,9]. The maximal difference acceptable for the AS+AQ to be considered as clinically non-inferior is 10% (absolute value in percentage) [10,11]. For a statistical power of 80% (β = 20%) a risk α = 5% and using 95% confidence level sample size for each arm was 207. In order to prevent premature stops, this number was increased to 10% given a total of 456 patients (76 patients per arm in each country). Data were entered using Epi info software version 6.04d. All analysis was done with STATA IC 10™ software.

An intention to treat (ITT) and per protocol (PP) analysis were done. ITT included all randomized participants who took at least one full dose and had one post baseline efficacy assessment without major protocol violations as wrong dosage, wrong use of non-assigned drug by mistake, co-infection with other malaria species. Patients with major violations and patients lost during the follow-up or withdrawn (due to an adverse event or to the use of another drug with anti-malarial activity or withdrawal consent) have been considered as failure. The per protocol analysis included patients who received the three doses and who had no major protocol violation up to the day 28. Those lost to follow up, and the withdrawals of consent were excluded from the per protocol analysis.

The primary endpoint was the Adequate Clinical and Parasitological Response (ACPR) which is an absence of parasitaemia on Day 28 irrespective of axillary temperature without previously meeting any of the criteria of Early Treatment Failure or Late Clinical Failure [4]. The cumulative incidence of failure rate was calculated in each study arm and compared using Kaplan-Meir method. The secondary endpoints were the comparison of fever and parasites clearances, gametocytes carriage after treatment and tolerability of the two regimens.

Data were analysed by estimations of difference in proportions corresponding to 95% confidence interval. Comparison between groups was made using chi-square test or Fisher exact test for qualitative outcome and student test for quantitative outcome when applicable. Otherwise, non-parametric tests (Man Withney, Kruskall Wallis) were used. A p value (two-sided) less than 0.05 was considered statistically significant.

### Ethical considerations

The protocol was reviewed and approved by the national ethical committees of each country.

## Results

### Trial profile

**Figure 1:**
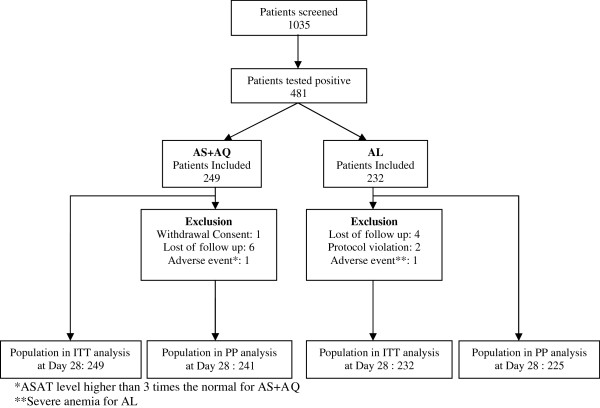
Trial profile.

In 1,035 patients screened, 481 were included: 249 in AS+AQ arm and 232 in AL arm (Figure 1). Fifteen patients were excluded: 10 lost during the follow up, one following withdrawal of consent, two for protocol violation and two patients for high ALAT and ASAT. At the end of the study, 481 patients were included in the ITT analysis and 466 patients in PP analysis.

### Demographics characteristics baseline

The two populations were similar regarding age mean (age mean, weight mean and sex ratio). Any difference was noted regarding fever and parasitaemia mean (Table 1).

**Table 1:** Demographics characteristics baseline of patients included

**Parameters**	**AS-AQ**	**AL**	**p value**
Age (median, range)	4 (1 – 7 years)	5 (1 – 7 years)	0.22
Sex ratio (M/F)	1.2	0.9	0.27
Weight mean (kg)	15.5 ± 6.4	16.5 ± 3.7	0.24
Axillary temperature mean (°C)	38.8 ± 0.8	38.7 ± 0.8	0.99
Parasite density Geometric mean (P/μl)	11952	13212	0.32

± standard deviation.

### Patient’s biological characteristics at inclusion

Any significant difference was noted between the two groups regarding the hemoglobin, creatinine and liver enzymes means (Table 2).

**Table 2:** Biological parameters evolution between day 0 and day 7

**Parameters**	**AS-AQ**			**AL**		
	**D0**	**D7**	**p**	**D0**	**D7**	**p**
Mean Hb level (g/dl)	9,5 ± 2	9.3 ± 1.5	0.75	10 ± 2	9.6 ±16	0.63
Anemia	71,9	87,1	<10^-3^	66,4	78,9	0,002
(Hb < 11g/dl) (%)						
Patient with abnormal	8.4	4.4	0,05	7.3	6	0.25
ALAT (<40 UI/L) (%)						
Patient with abnormal	30.1	10	<10^-3^	27.6	12.9	<10^-3^
ASAT (<40 UI/L) (%)						
Mean bilirubine level	11.2 ±8.5	7.3 ±3.6	0.58	11.8 ± 11.4	7.4 ±3.4	0.55
Mean Creatinin level	5.8 ±2.4	5.9 ±2.5	0.99	6 ±2.2	5.8 ± 2.3	0.85

### Therapeutic efficacy

**Figure 2:**
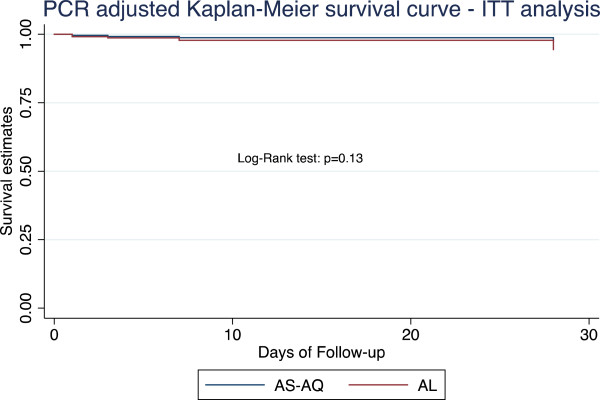
Kaplan-Meir survival estimates of PCR adjusted ACPR by treatment arm – ITT analysis.

The ACRP at D28 after PCR correction in ITT analysis was 97.6% for AS+AQ versus 94.8% for AL (difference 0.027 95% CI [-0.006; 0.062]) (p= 0.11). In PP analysis, it was 98.7% in AS+AQ arm versus 96.9% in AL arm (difference 0.18 95% CI [-0.007; 0.044] (p= 0.16). Thus, the non-inferiority of AS+AQ was shown both in intention-to-treat and per-protocol analysis. Regarding treatment failure, after PCR correction, 1.6% (4 patients) in AS+AQ group and 3.9% (9 patients) in AL group were considered as recrudescent (Table 3). Any difference was obtained in the therapeutic efficacy analysis done per country (Table 4). The Kaplan Meier analysis didn’t show any significant difference between the two group in terms of incidence of failure rate (log rank test: p= 0.12) (Figure 2).

**Table 3:** Treatment outcome of AS+AQ versus AL at D28

	**Artesunate/Amodiaquine**	**Artemether/Lumefantrine**	**p value**
**Overall treatment outcome**			
**ITT analysis**			
Early treatment failure	00	00	--
Crude parasitological failure at day 28	05/249 (2%)	10/232 (4.3%)	0.14
PCR adjusted failure rate at day 28	04/249 (1.6%)	09/232 (3.8%)	0.12
PCR adjusted ACPR at day 28	242/249 (97.6%)	218/232 (94.8%)	0.11
Difference, % [95%CI] 2.7 [-0.6 - 6.2%]			
**PP analysis**			
Early treatment failure	00	00	--
Crude parasitological failure at day 28	05/246 (2%)	10/227 (4.4%)	0.14
PCR adjusted failure rate at day 28	04/246 (1.6%)	09/227 (3.9%)	0.12
PCR adjusted ACPR at day 28	242/246 (98.7%)	218/227 (96.9%)	0.16
Difference, % [95%CI] 1.8 [-0.76 - 4.4]			

**Table 4:** Treatment outcome stratified by country

**Treatment outcome stratified by country**	**Artesunate/Amodiaquine**	**Artemether/Lumefantrine**	**p value**
**Senegal (160 patients)**	**80**	**80**	
**ITT analysis**			
Early treatment failure	00	00	--
Crude Parasitological failure at day 28	01/88 (1.1%)	01/72 (1.4%)	1
PCR adjusted failure rate at day 28	01/88 (1.1%)	01/72 (1.4%)	1
PCR adjusted ACPR at day 28	85/88 (96.6%)	67/72 (93.1%)	0.55
% difference	0.3 [-3.3;3.9]		
**PP analysis**			
Early treatment failure	00	00	--
Crude Parasitological failure at day 28	01/86 (1.2%)	01/68 (1.5%)	1
PCR adjusted failure rate at day 28	01/86 (1.2%)	01/68 (1.5%)	1
PCR adjusted ACPR at day 28	85/86 (98.8%)	67/68 (98.5%)	0.87
% difference	0.3 [-3.3;3.9]		
**Ivory Coast (160 patients)**	**80**	**80**	
**ITT analysis**			
Early treatment failure	00	00	--
Crude Parasitological failure at day 28	02/80 (2.5%)	04/80 (2.5%)	0.68
PCR adjusted failure rate at day 28	01/80 (1.3%)	02/80 (2.5%)	1
PCR adjusted ACPR at day 28	79/80 (98.8%)	77/80 (96.3%)	0.36
% difference	2.5 [-0.9;5.9]		
**PP analysis**			
Early treatment failure	00	00	
Crude Parasitological failure at day 28	02/79 (2.5%)	04/79 (5.1%)	0.68
PCR adjusted failure rate at day 28	01/79 (1.2%)	02/79 (2.5%)	1
PCR adjusted ACPR at day 28	79/79 (100%)	77/79 (97.5%)	0.16
% difference	2.5 [0.9;5.6]		
**Cameroun (161 patients)**	**81**	**80**	
**ITT analysis**			
Early treatment failure	00	00	
Crude Parasitological failure at day 28	02/81 (2.5%)	04/80 (5%)	0.66
PCR adjusted failure rate at day 28	02/81 (2.5%)	04/80 (5%)	0.66
PCR adjusted ACPR at day 28	79/81 (97.5%)	76/80 (95%)	0.40
% difference, 95%CI	2.5% [-3.3;8.4]		
**PP analysis**			
Early treatment failure	00	00	--
Crude Parasitological failure at day 28	02/81 (2.5%)	04/80 (5%)	0.66
PCR adjusted failure rate at day 28	02/81 (2.5%)	04/80 (5%)	0.66
PCR adjusted ACPR at day 28	79/81 (97.5%)	76/80 (95%)	0.40
% difference	2.5% [-3.3;8.4]		

### Fever and parasite clearance

**Figure 3:**
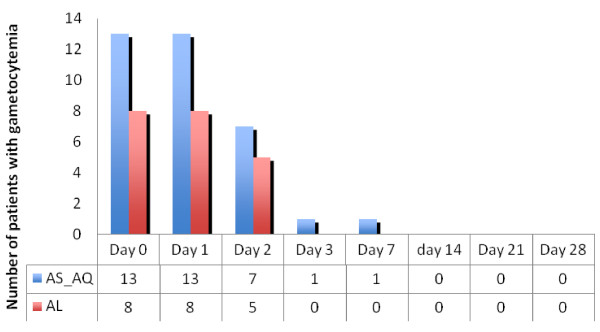
Patients carrying gametocytes from day 0 to day 28 in the two groups.

A rapid decrease of fever was noted during the follow-up in the two groups. At day 0, all patients were feverish. At day 1, 39% in Al group and 34.7% in AS+AQ group presented a fever. At day 2, the percentage of patients with fever decrease to 4.8% and 2.8% respectively in Al and AS+AQ groups. At day 3, the fever clearance was total in all the patients. Parasite clearance was faster in the two groups. At day 2, only 4.8% in AL group and 2% in AS+AQ presented an asexual parasite stage carriage. At day 3, the parasite clearance was total. Regarding the gametocyte carriage, at the inclusion, 13% and 31%, respectively in AS+AQ and AL arms, presented gametocytes. The evolution showed a total clearance starting from day 1 in AL group, while in AS-AQ group, a persistence of gametocytes until day 3 was noted (Figure 3).

### Clinical and biological tolerance

In the two groups, the clinical tolerance was good (Table 4). Thirty-three patients (13.2%) in AS+AQ group and fourteen patients in AL group presented at least one adverse event between day 1 and day 28. Two patients presented a severe adverse event requiring their exclusion of the study: one in AS+AQ group with an increase of ASAT with a rate higher than three times the normal at day 7 and one in AL group presenting a severe anaemia, with a rate of haemoglobin less than 5g/dl at day 7. The evolution at day 28 was favourable for these two patients.

In the two groups, a significant increase of patients with anaemia (Hb < 11g/dl) [12] between day 0 and day 7 was observed. This was more important in the AS+AQ group (Table 2). Regarding the hepatic function, an improvement was noted in the two groups with a significant difference for ASAT. Any significant statistical variation was found for bilirubin and creatinine levels.

## Discussion

This multicentre study allowed an evaluation of the efficacy and tolerance of AS+AQ versus AL in three countries, (Cameroon, Ivory Coast and Senegal) in the treatment of uncomplicated *Plasmodium falciparum* malaria among children between six months and 7 years of age. The results obtained show that the AS+AQ association proved to be very effective with a ACPR rate adjusted by 97.2% in ITT and 98.3% in PP. Various studies conducted with artesunate/amodiaquine free combination, showed comparable efficacies with a non-inferiority to AL [13] and other formulations of ACT (artesunate/sulphadoxine-pyrimethamine, dihydroartemisinine/piperaquine, artesunate/chlorproguanil-Dapsone) [14-18]. This new paediatric formulation administered once a day, besides reducing the number of doses (usually twice a day) confirms the efficacy of this association [19]. Similarly, this paediatric formulation complies with dosages recommended by WHO, which allow an efficacy optimization [20]. However, it should be pointed out that this good efficacy is highly correlated with a supervised dose administration. In fact, several studies demonstrated a lower efficacy level in case of a non-supervised administration [21-23]. This drop in compliance when the association is used in daily practice (non-supervised dose) may in the long-term result in reduction of efficacy and could lead to the selection of resistant strains [21].

Fever clearance was fast in both treatment groups, confirming the previous data [8]. A rapid disappearance of asexual parasites in the blood has been observed in both treatment groups with a total body clearance from day 3. PCR demonstrated a lower recrudescence level in the AS+AQ group without any significant difference (p= 0.12). However, gametocyte carriage was longer in the AS+AQ group with a complete disappearance from D7. A rapid efficacy of this association on gametocyte carriage has been demonstrated [24], allowing a reduction in the *P. falciparum* transmission.

The clinical tolerance was good with minor adverse events such as asthenia and vomiting in both treatment groups. Administering a single daily dose of this new formulation seems to be well-tolerated and confirms the previous results [19]. Regarding biological aspects, a significant increase in the number of patients presenting an anaemia has been observed in both groups. This confirms the results obtained by Sowunmi *et al* showing an increase in anaemia, especially after treatment with the AS+AQ association [25]. However, the same results obtained with the AL association seem to suggest another cause different from the amodiaquine effect. Two serious biological adverse events have been observed; one in each group at day 7 with a good evolution at day 28. The liver function has been improved significantly in treatment groups.

## Conclusion

This study shows that the new formulation in a suspension form of the artesunate/amodiaquine association is as effective as artemether/lumefantrine in the treatment of uncomplicated *P. falciparum* malaria in the African child. The single daily dose may help improve compliance when used in daily practice with non-supervised administration.

## Competing interests

The investigations were financially supported by Pfizer Pharmaceutical Laboratories, West Africa, who also supplied the anti-malarials drugs used for this study.

## Authors’ contribution

BF wrote the paper; BF, JLN, RCT, KS supervised field work and data collection in Senegal, analysed data of Senegal and analysed from the three countries, reviewed and approved the manuscript; TK, TN collected and analysed data in Cameroon, reviewed and approved the manuscript; EIHM, CPK, CA A collected and analysed data in Ivory Coast, reviewed and approved the manuscript; OG coordinated the study in the three countries, reviewed and approved the manuscript; OF coordinated the study in Senegal, reviewed and approved the manuscript; AS coordinated the study in Cameroon (data collection and analysis) reviewed and approved the manuscript; MK coordinated (data collection and analysis) the study in Ivory Coast, reviewed and approved the manuscript. All authors read and approved the final manuscript.
